# Chemotherapy for metastatic soft tissue sarcomas--another full circle?

**DOI:** 10.1038/bjc.1991.230

**Published:** 1991-07

**Authors:** V. H. Bramwell


*Br. J. Cancer* (1991), **64**, 7–9 © Macmillan Press Ltd., 1991

**GUEST EDITORIAL**

### Chemotherapy for metastatic soft tissue sarcomas – another full circle?

V.H.C. Bramwell

*Department Oncology, University of Western Ontario, London Regional Cancer Centre, 790 Commissioners Road, East London, Ontario N6A 4L6, Canada.*

After attending the sarcoma session of the 1990 meeting of the American Society for Clinical Oncology, one might be forgiven for wondering if chemotherapy for metastatic soft tissue sarcoma (STS) has come a full circle. It is 18 years since the South West Oncology Group (SWOG) published results of the first large scale study of doxorubicin (ADR) (O'Bryan *et al.*, 1973). In 45 evaluable patients there were 13 complete and partial responses, giving an overall response rate of 29%. Since that time a variety of combination chemotherapy regimens have been tested, with overall response rates as high as 71% (Bodey *et al.*, 1981). Yet, in the largest randomised phase III trial ever performed in metastatic STS (Santoro *et al.*, 1990) the European Organisation for Research and Treatment of Cancer (EORTC) has failed to demonstrate superiority for combination chemotherapy, based on cyclophosphamide or ifosfamide, compared with doxorubicin alone. Why should this be, and does it mean we have reached an impasse in the management of this disease, once it is disseminated?

SWOG explored the addition of other agents to doxorubicin in a logical sequence of studies (Gottlieb *et al.*, 1975), but individual regimens were not compared with each other in randomised trials. The rationale for adding dacarbazine (DIC) was its proven, although limited activity as a single agent (Gottlieb *et al.*, 1976). However, the addition of cyclophosphamide (CYCLO) and vincristine (VCR) was purely speculative based on the efficacy of these drugs in childhood sarcomas.

This series of studies (Gottlieb *et al.*, 1975) showed respective response rates of 31%, 42%, 42% and 55% for ADR alone, ADIC, VADIC and CYVADIC (CYCLO + VCR + ADR + DIC). Exclusion of bone tumours from the CYVADIC study left a total of 118 patients with STS, in whom the response rate was 59%. Since then, despite marginal activity as single agents, other drugs such as actinomycin D (Benjamin *et al.*, 1976; Schoenfeld *et al.*, 1982), cisplatinum (Edmonson *et al.*, 1983, 1985; Cormier *et al.*, 1986) and methotrexate (Bryant *et al.*, 1980; Lynch *et al.*, 1982; Presgrave *et al.*, 1987) have been incorporated into ADR based combinations. Although there were insignificant differences in response rate and survival when actinomycin D (DACT) was substituted for DIC in CYVADIC (Benjamin *et al.*, 1976) in a subsequent randomised trial of SWOG, other combinations including DACT had limited activity (Schoenfeld *et al.*, 1982). Thus, in the late 1970's CYVADIC became a standard treatment, and was the subject of at least ten large scale studies (Bramwell, 1988).

Unfortunately other investigators were unable to reproduce the results achieved by SWOG for the CYVADIC combination. Later response rates ranged from 20% (Giuliano *et al.*, 1978) through 27% (Karakousis *et al.*, 1982) and 39% (Pinedo *et al.*, 1985) to 48% (Bui *et al.*, 1985). In 1980, Yap *et al.* reanalysed the SWOG data set from the first CYVADIC study (Gottlieb *et al.*, 1976). For 125 patients with STS deemed to be eligible, the overall response rate was somewhat lower at 50%. Many investigators started to question the contribution of CYCLO and VCR, and there was a move back to ADR/DIC combinations, which permitted the use of a slightly higher dose of ADR (60 mg $m^{-2}$ *vs* 50 mg $m^{-2}$ in CYVADIC). Surprisingly ADIC has never been compared directly with CYVADIC in a large scale randomised trial.

ECOG carried out one of the earliest randomised studies evaluating the efficacy of high dose ADR (70 mg $m^{-2}$ every 3 weeks), in comparison with two combination chemotherapy regimens (Schoenfeld *et al.*, 1982). ADR was statistically superior to VAC (VCR + DACT + CYCLO) with respective response rates of 27% and 11%, confirming the lack of efficacy of DACT containing combinations in adult STS. Although the response rate for single agent ADR was higher than the 19% achieved with the second combination regimen (VCR + ADR + CYCLO) used in this study, the difference was not significant. A later trial by SWOG, randomising 335 patients (Baker *et al.*, 1987), showed that adding CYCLO or DACT to the ADIC regimen did not significantly improve response rates, which were respectively 33% (ADIC), 34% (CYCLO/ADIC) and 24% (DACT/ADIC).

ECOG performed another three arm trial (Bordon *et al.*, 1987) in which patients were randomised to high dose ADR as described above (Schoenfeld *et al.*, 1982); or to ADR 20 mg $m^{-2}$ d1,2,3 followed by 15 mg $m^{-2}$ d8 and weekly thereafter; or to ADIC. The response rate to ADIC was significantly better at 30% ($P<0.02$), compared with 18% and 16% for the 3 weekly and loading/weekly dose schedules of ADR, although complete response rates, durations of response and survival were similar for the three regimens. The authors' conclusion questioning 'whether the increased response frequency, without an impact on survival, is worth the significantly greater toxicity' was criticised in an accompanying editorial (Benjamin, 1987), which suggested that higher doses of ADIC incorporating Ifosfamide (IFOS) initially or subsequently should be pursued. Another interesting point, not commented on in the paper or editorial is that the response rate in this second ECOG study, to the 3 weekly schedule of ADR was low compared with the previous study, 18% *vs* 27%, although the dose/schedule was identical. Because of variability in patient populations, direct comparisons between studies have limited value. However, a response rate of 27% is in keeping with many other studies of intermittent high dose ADR (Bramwell *et al.*, 1983; Mouridsen *et al.*, 1987; Santoro *et al.*, 1990), and is similar to the respose rate for ADIC (30%) in this second ECOG trial (Bordon *et al.*, 1987) and for ADIC (33%) in the SWOG trial (Baker *et al.*, 1987).

Benjamin's editorial (1987) suggested that 'IFOS intensification' might produce better results. IFOS had been synthesised in Germany in the 1960's, and shown to have a broad spectrum of activity, but a high incidence of haemorrhagic cystitis had discouraged its widespread use. The discovery of Mesna, a sulphydryl donor, which neutralises the toxic byproduct acrolein in the bladder, led to a re-evaluation of IFOS in the 1980's, and promising results were reported

Received and accepted: 11 February 1991.

for STS (Stuart-Harris *et al.*, 1983; Bramwell *et al.*, 1986; Antman *et al.*, 1989). Preliminary data suggested that response rates between 25–35% could be achieved in patients not previously exposed to chemotherapy. Such activity was higher than that seen for any single agent other than ADR. A randomised study (Bramwell *et al.*, 1986) conducted by the EORTC demonstrated that IFOS $5\,g\,m^{-2}$ q 3 weeks was more active than CYCLO $1.5\,g\,m^{-2}$, but less myelosuppressive and could be incorporated without dose reduction into combination chemotherapy with $50-60\,mg\,m^{-2}$ ADR.

US investigators chose to incorporate IFOS into the ADIC combination, and early results for a high dose 96 h infusional regimen, MAID, were extremely promising (Elias *et al.*, 1989), with an overall response rate of 47% in 108 evaluable patients. European investigators omitted DIC and concentrated on ADR + IFOS combinations, often based on a more convenient 36 h infusional schedule of IFOS/Mesna. Response rates were somewhat lower, 22–38% (Mansi *et al.*, 1988; Cantwell *et al.*, 1988; Schutte *et al.*, 1990) but in the same range as previous European experience for combination chemotherapy in STS.

The critical test of these new IFOS combinations is direct comparison with standard regimens such as ADR, ADIC and CYVADIC, and the American Intergroup and the EORTC have completed the appropriate studies. Preliminary results (Antman *et al.*, 1990) of the Intergroup trial comparing ADIC with MAID, have shown no differences in response rates or survival between the two arms. To date, this study has only appeared in abstract form, with 268 of the 421 patients entered currently evaluable, and actual response rates were not quoted. The EORTC study (Santoro *et al.*, 1990), which accrued 716 patients, reported response rates of 24% for ADR, 27% for ADR + IFOS and 28% for CYVADIC, based on 549 patients currently evaluable. These results were not significantly different, and curves of time to relapse and survival overlapped. Combination chemotherapy was significantly more toxic.

It is tempting to conclude from these data that single agent ADR in doses of $\geqslant 70\,mg\,m^{-2}$ q 3 weeks should be the standard treatment for patients wishing to receive palliative chemotherapy outside a clinical trial. Prolonged infusions, splitting the dose over several days, or weekly treatment, may reduce toxicity but add to the inconvenience.

A corollary of this conclusion would be the recommendation that promising new treatments should be compared, at the earliest opportunity, with high dose single agent ADR in a randomised phase III setting.

In the research setting, should we continue to explore other avenues, or are these results so depressing that we should abandon the intense investigation of these rare tumours? I remain optimistic that progress can be made. Strategies should include:

(a) identification of new active agents, preferably with novel structures and different modes of action and toxicity;
(b) synthesis of analogues of current effective agents, especially if the structural changes might enhance activity;
(c) definition of improved/dose schedules which might optimise activity particularly for new agents such as IFOS;
(d) dose intensification which may be permitted by growth factor support and
(e) novel approaches, exploiting discoveries in the rapidly expanding field of molecular biology which improve our understanding of patterns of failure and mechanisms of drug resistance.

Studies are currently underway in most of these areas. Drugs with novel structures should be tested in STS, although the smaller potential market may not make this a top priority for industry sponsored trials. Analogues of anthracyclines and alkylating agents are obvious candidates for testing in STS, but anti-folates are an additional interesting group. Early reports (Subramanian & Wiltshaw, 1978) of significant activity for MTX have not been substantiated (Presgrave *et al.*, 1987; Pfeffer *et al.*, 1988), but the Canadian Sarcoma Group (CSG) has reported that trimetrexate has modest activity in previously untreated patients (Quirt *et al.*, 1988). Unfortunately, it seems unlikely that this drug will be developed as an anticancer agent because of its limited activity in other tumour types and unpredictable myelotoxicity. Another promising anti-folate, 10-EDAM, is currently being studied by the CSG. The limited efficacy of current single agent and combination chemotherapy in STS, makes it entirely feasible to propose single agent investigational drug therapy to patients as first line therapy, with the option to proceed to standard ADR, alone or in combination, if this fails. German groups (Hilgard *et al.*, 1983; Brade *et al.*, 1985) have led the field in exploring high and prolonged dose/schedules of IFOS, but comparative studies are lacking.

Strategy (d) has been pursued by the EORTC, which has used GM-CSF to permit dose intensification of ADR + IFOS, escalating ADR to $75\,mg\,m^{-2}$. Early results (unpublished) are promising, and a randomised trial comparing this dose escalated regimen with standard dose ADR + IFOS will soon be initiated. This approach has similarities to that reported by Bodey *et al.* in 1981, who administered escalated doses of CYVADIC in the setting of a protected environment-prophylactic antibiotic program. In a small group of patients, a higher response rate (71%) was achieved and, despite profound myelosuppression, infective episodes were no more frequent than with standard therapy. Although it achieved its objectives, the inconvenience and costs of such a program in a palliative setting have limited its use. It remains to be seen whether GM-CSF regimens will prove to be of benefit, and cost-effective in this setting.

There have been many interesting findings relevant to STS in the field of molecular biology. Specific chromosomal breaks have been associated with particular subtypes of sarcomas (Karakousis *et al.*, 1987), over expression of the MDR-1 gene may be linked with resistance to certain chemotherapeutic drugs (Gerlach *et al.*, 1987; Chan *et al.*, 1990) and other mechanisms of resistance are being explored. Different levels of oncogene expression may have a bearing on the behaviour of STS (Fahrer *et al.*, 1989; Kato *et al.*, 1990) and further elucidation of these patterns might gives us clues to alternative treatment strategies.

In conclusion, identification of substantially more active drugs or combination chemotherapy will provide not only a better means of palliating metastatic disease, but might also have the potential to cure patients if used appropriately as adjuvant treatment.

### References

ANTMAN, K., BAKER, L., BALCERZAK, S. & CROWLEY, J. (1990). An intergroup (SWOG + CALGB) phase III randomized study of doxorubicin + dacarbazine with or without ifosfamide + Mesna in advanced soft tissue and bone sarcomas. *Proc. Am. Soc. Clin. Oncol.*, **9**, 311.

ANTMAN, K.H., RYAN, L., ELIAS, A., SHERMAN, D. & GRIER, H.E. (1989). Response to Ifosfamide and Mesna: 124 previously treated patients with metastatic or unresectable sarcoma. *J. Clin. Oncol.*, **7**, 126.

BAKER, L.H., FRANK, J., FINE, G. & 6 others (1987). Combination chemotherapy using adriamycin, DTIC, cyclophosphamide, and actinomycin D for advanced soft tissue sarcomas: a randomized comparative trial. A phase III Southwest Oncology Group Study (7613). *J. Clin. Oncol.*, **5**, 851.

BENJAMIN, R.S. (1987). Editorial: grade 3 nausea, vomiting and myelosuppression or progressive, metastatic sarcoma. *J. Clin. Oncol.*, **5**, 833.

BENJAMIN, R.S., GOTTLIEB, J.A., BAKER, L.O. & SINKOVICS, J.G. (1976). CYVADIC *vs* CYVADACT: a randomized trial of cyclophosphamide, vincristine and adriamycin + DTIC or actinomycin D in metastatic sarcomas. *Proc. Am. Assoc. Cancer Res.*, **17**, 256.

BODEY, G.P., RODRIGUEZ, V., MURPHY, W.K., BURGESS, A. & BENJAMIN, R.S. (1981). Protected environment-prophylactic antibiotic program for malignant sarcomas: randomized trial during remission induction chemotherapy. *Cancer*, **47**, 2422.

BORDEN, E.C., AMATO, D.A., ROSENBAUM, C. & 5 others (1987). Randomized comparison of three adriamycin regimens for metastatic soft tissue sarcomas. *J. Clin. Oncol.*, **5**, 840.

BRADE, W.P., HERDRICH, K. & VARINI, M. (1985). Ifosfamide – pharmacology, safety and therapeutic potential. *Cancer Treat. Rev.*, **12**, 1.

BRAMWELL, V.H.C. (1988). Symposium on current perspectives in the management of soft tissue sarcomas. 2. The role of chemotherapy in multi-modality therapy. *Can. J. Surg.*, **31**, 390.

BRAMWELL, V.H.C., MOURIDSEN, H.T., MULDER, J.H. & 6 others (1983). Carcinomycin *vs* adriamycin in advanced soft tissue sarcomas: an EORTC randomized phase II study. *Eur. J. Cancer Clin. Oncol.*, **19**, 1097.

BRAMWELL, V.H.C., MOURIDSEN, H.T., SANTORO, A. & 8 others (1986). Cyclophosphamide *vs* ifosfamide: final report of a randomized phase II trial in adult soft tissue sarcomas. *Eur. J. Cancer Clin. Oncol.*, **23**, 311.

BRYANT, B.M. & WILTSHAW, E. (1980). Results of the Royal Marsden Hospital second soft tissue sarcoma schedule (STS II) chemotherapy regimen in the management of advanced sarcoma. *Cancer Treat. Rep.*, **64**, 689.

BUI, N.B., CHAUVERGNE, J., HOCKE, C., DURAND, M., BRUNET, R. & COINDRE, J.M. (1985). Analysis of a series of sixty soft tissue sarcomas in adults treated with a cyclophosphamide-vincristine-adriamycin-dacarbazine (CYVADIC) combination. *Cancer Chemother. Pharmacol.*, **15**, 82.

CANTWELL, B.M.J., CARMICHAEL, J., GHANI, S. & HARRIS, A.L. (1988). A phase II study of ifosamide/Mesna with doxorubicin for adult soft tissue sarcoma. *Cancer Chemother. Pharmacol.*, **21**, 49.

CHAN, H.S.L., THORNER, P.S., HADDAD, G. & LING, V. (1990). Immunohistochemical detection of P-glycoprotein: prognostic correlation in soft tissue sarcoma of childhood. *J. Clin. Oncol.*, **8**, 689.

CORMIER, W.J., HAHN, R.G., EDMONSON, J.H. & EAGAN, R.T. (1986). Phase II study of advanced sarcoma: randomized trial of pyrazofurin versus combination cyclophosphamide, doxorubicin and cis-Dichlorodiammine platinum (II) CAP. *Cancer Treat. Rep.*, **64**, 655.

EDMONSON, J.H., HAHN, R.G., SCHUTT, A.J., BISEL, H.F. & INGLE, J.N. (1983). Cyclophosphamide, doxorubicin and cisplatin combined in the treatment of advanced sarcomas. *Med. Paed. Oncol.*, **11**, 319.

EDMONSON, J.H., LONG, H.J., RICHARDSON, R.L., CREAGAN, E.T. & GREEN, S.J. (1985). Phase II study of a combination of mitomycin, doxorubicin and cisplatin in advanced sarcomas. *Cancer Chemother. Pharmacol.*, **15**, 181.

ELIAS, A., RYAN, L., SULKES, A., COLLINS, J., AISNER, J. & ANTMAN, K.H. (1989). Response to Mesna, doxorubicin, ifosfamide and dacarbazine in 108 patients with metastatic or unresectable sarcoma and no prior chemotherapy. *J. Clin. Oncol.*, **7**, 1208.

FAHRER, C., BRACHMANN, R. & VON DER HELM, K. (1989). Expression of c-sis and other cellular proto-oncogenes in human sarcoma cell lines and biopsies. *Int. J. Cancer*, **44**, 652.

GERLACH, J.H., BELL, D.R., KARAKOUSIS, C. & 5 others (1987). P-glycoprotein in human sarcoma: evidence for multidrug resistance. *J. Clin. Oncol.*, **5**, 1452.

GIULIANO, A.E., LARKIN, K.L., EILBER, F.R. & MORTON, D.L. (1978). Failure of combination chemotherapy (CYVADIC) in metastatic soft tissue sarcomas: implications for adjuvant studies. *Proc. Am. Soc. Clin. Oncol.*, **19**, 259.

GOTTLIEB, J.À., BAKER, L.H., O'BRYAN, R.M. & 15 others (1975). Adriamycin (NSC-123127) used alone and in combination for soft tissue and bone sarcomas. *Cancer Chem. Rep.*, **6**, 271.

GOTTLIEB, J.A., BENJAMIN, R.S., BAKER, L.H. & 16 others (1976). Role of DTIC (NSC-45388) in the chemotherapy of sarcomas. *Cancer Treat. Rep.*, **60**, 199.

HILGARD, P., HERDRICH, K. & BRADE, W. (1983). Ifosfamide – current aspects and perspectives. *Cancer Treat. Rev.*, **10** (Suppl A), 183.

KARAKOUSIS, C.P., CIN, P.D., TURC-CAREL, C., LIMON, J. & SANDBERG, A.A. (1987). Chromosomal changes in soft tissue sarcomas. A new diagnostic parameter. *Arch. Surg.*, **122**, 1257.

KARAKOUSIS, C.P., RAO, U. & PARK, H.C. (1982). Combination chemotherapy (CYVADIC) in metastatic soft tissue sarcomas. *Eur. J. Cancer Clin. Oncol.*, **18**, 33.

KATO, M., TOGUCHIDA, J., HONDA, K. & 7 others (1990). Elevated frequency of a specific allele of the L-MYC gene male patients with bone and soft tissue sarcomas. *Int. J. Cancer*, **45**, 47.

LYNCH, G., MAGILL, G.B., SORDILLO, P. & GOLBEY, R.B. (1982). Combination chemotherapy of advanced sarcomas in adults with 'CYOMAD' (S7). *Cancer*, **50**, 1724.

MANSI, J.L., FISHER, C., WILTSHAW, E., MACMILLAN, S., KING, M. & STUART-HARRIS, R. (1988). A phase I-II study of ifosfamide in combination with adriamycin in the treatment of adult soft tissue sarcoma. *Eur. J. Cancer Clin. Oncol.*, **24**, 1439.

MOURIDSEN, H.T., BASTHOLT, L., SOMERS, R. & 8 others (1987). Adriamycin versus epiadriamycin in advanced soft tissue sarcomas. A randomized phase II/phase III study of the EORTC Soft Tissue and Bone Sarcoma Group. *Eur. J. Cancer Clin. Oncol.*, **23**, 1477.

O'BRYAN, R.M., LUCE, J.K. & TALLEY, R.W. (1973). Phase II evaluation of adriamycin in human neoplasia. *Cancer*, **32**, 1.

PFEFFER, M.R., SULKES, A. & BIRAN, S. (1988). Cyclophosphamide, adriamycin, DTIC and vincristine with methotrexate in the treatment of advanced soft tissue sarcomas. *Inst. J. Med. Sci.*, **24**, 599.

PINEDO, H.M.. BRAMWELL, V.H.C., MOURIDSEN, H.T. & 11 others (1984). CYVADIC in advanced soft tissue sarcoma: a randomized study comparing two schedules. A study of the EORTC Soft Tissue and Bone Sarcoma Group. *Cancer*, **53**, 1825.

PRESGRAVE, P., WOODS, R.L., TATTERSALL, M.H. & 4 others (1987). Chemotherapy of adult soft tissue sarcoma with combination of doxorubicin and methotrexate. *Cancer Treat. Rep.*, **71**, 1087.

QUIRT, I., EISENHAUEER, E., KNOWLING, M. & 5 others (1988). A phase II study of trimetraxate in metastatic soft tissue sarcoma. *Proc. Am. Soc. Clin. Oncol.*, **7**, 275.

SANTORO, A., ROUESSE, J., STEWARD, W. & 10 others, for the EORTC Soft Tissue & Bone Sarcoma Group (1990). A randomized EORTC study in advanced soft tissue sarcomas (STS): ADM *vs* ADM + IFOS *vs* CYVADIC. *Proc. Am. Soc. Clin. Oncol.*, **9**, 309.

SHOENFELD, D.A., ROSENBAUM, C., HORTEON, J., WOLTER, J.M., FALKSON, G. & DECONTI, R.C. (1982). A comparison of adriamycin versus vincristine and adriamycin, and cyclophosphamide versus vincristine, actinomycin-D and cyclophosphamide for advanced sarcoma. *Cancer*, **50**, 2757.

SCHUTTE, J., MOURIDSEN, H.T., STEWART, W. & 8 others (1990). Ifosfamide plus doxorubicin in previously untreated patients with advanced soft tissue sarcoma. *Eur. J. Cancer*, **26**, 558.

STUART-HARRIS, R.C., HARPER, P.G., PARSONS, C.A. & 4 others (1983). High dose alkylation therapy using ifosfamide infusion with Mesna in the treatment of adult advanced soft tissue sarcoma. *Cancer Chem. Pharmacol.*, **11**, 69.

SUBRAMANIAN, S. & WILTSHAW, E. (1978). Chemotherapy of sarcoma. *Lancet*, **i**, 683.

YAP, B.S., BAKER, L.H., SINKOVICS, J.G. & 6 others (1980). Cyclophosphamide, vincristine, adriamycin and DTIC (CYVADIC) combination chemotherapy for the treatment of advanced sarcomas. *Cancer Treat. Rep.*, **64**, 93.


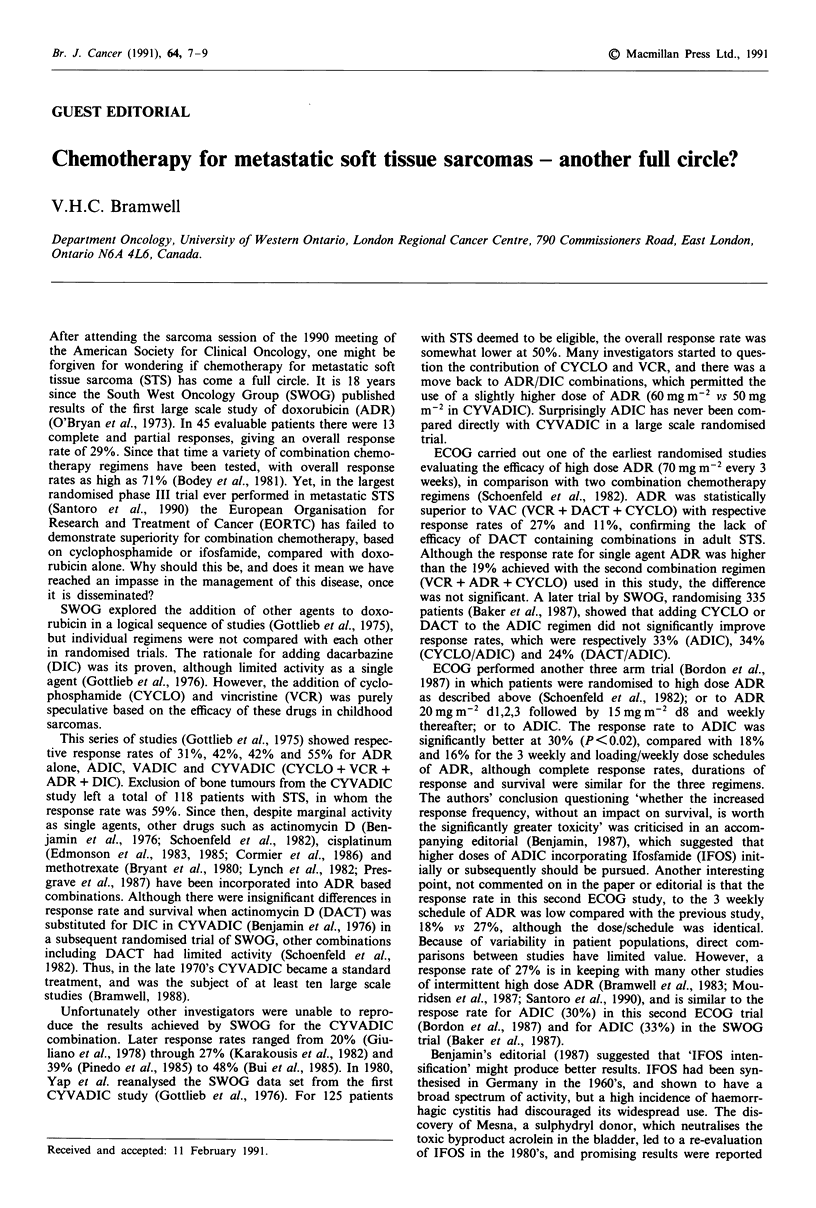

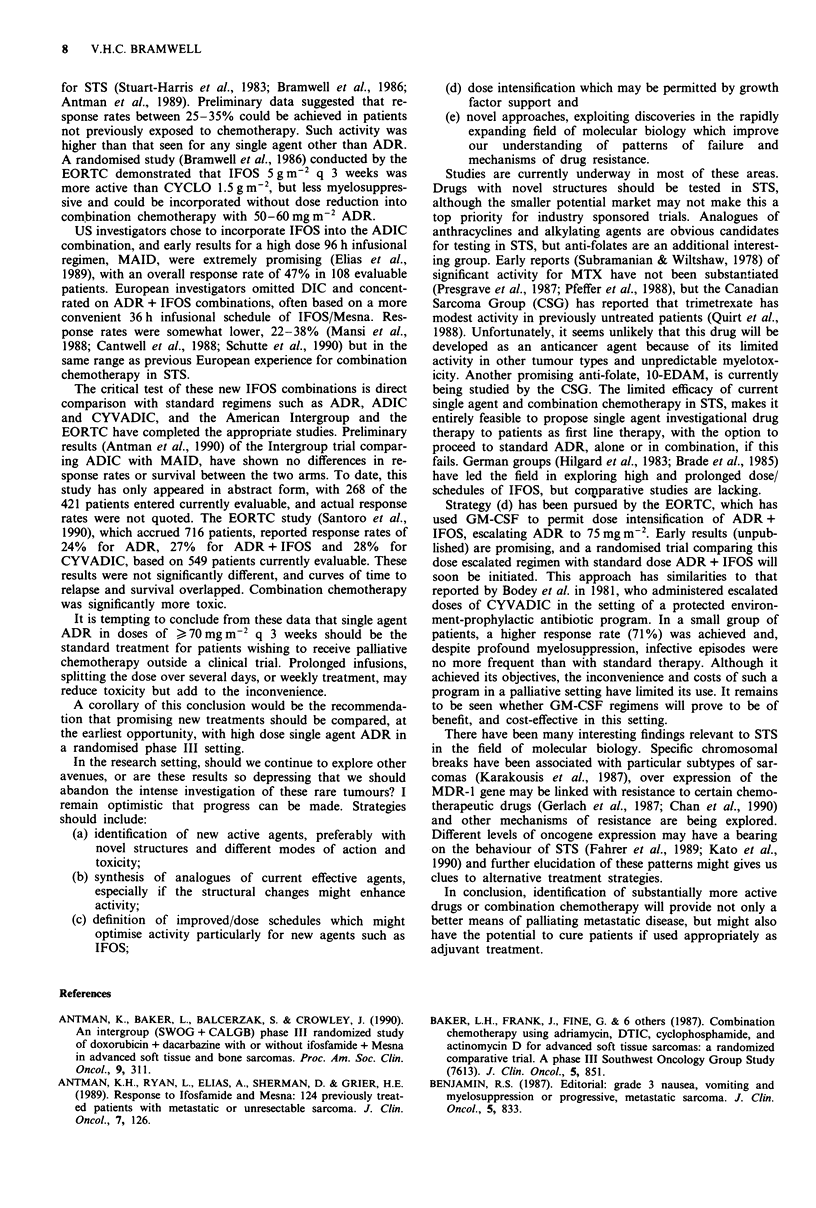

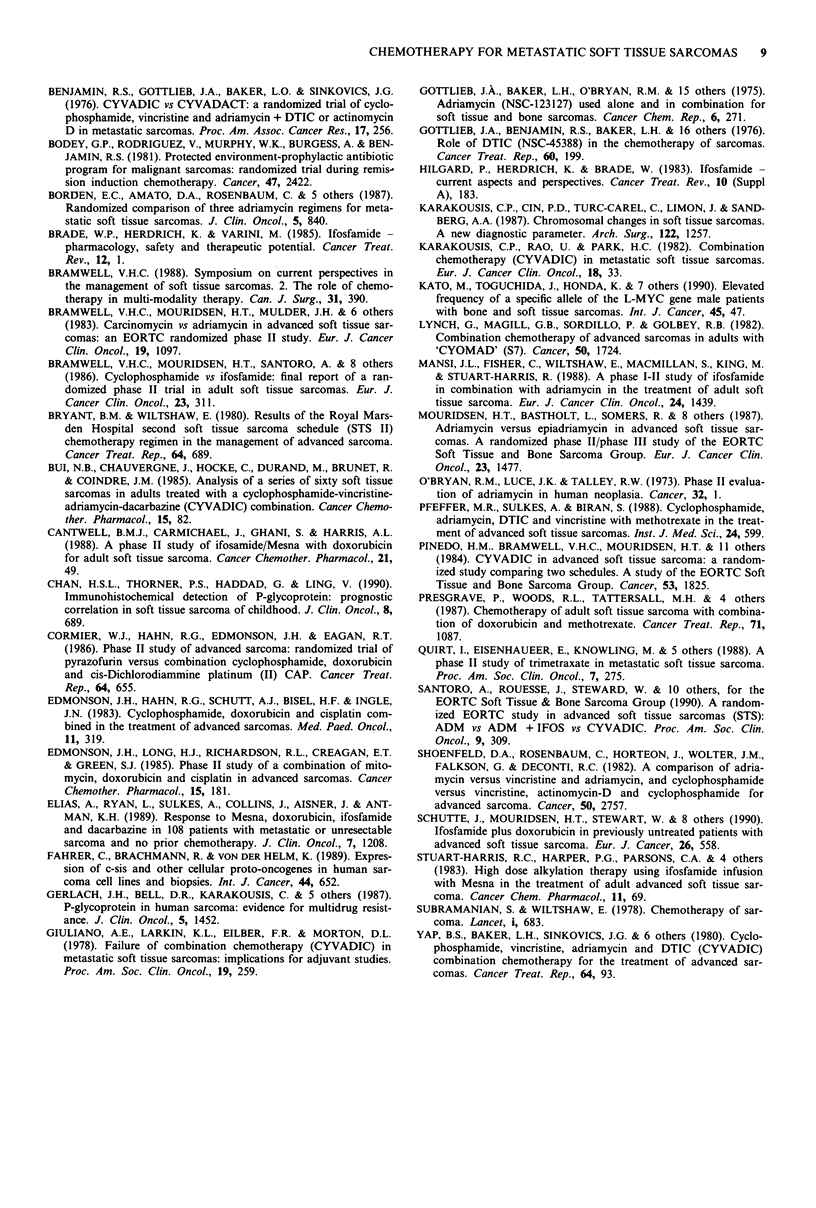


## References

[OCR_00292] Antman K. H., Ryan L., Elias A., Sherman D., Grier H. E. (1989). Response to ifosfamide and mesna: 124 previously treated patients with metastatic or unresectable sarcoma.. J Clin Oncol.

[OCR_00298] Baker L. H., Frank J., Fine G., Balcerzak S. P., Stephens R. L., Stuckey W. J., Rivkin S., Saiki J., Ward J. H. (1987). Combination chemotherapy using adriamycin, DTIC, cyclophosphamide, and actinomycin D for advanced soft tissue sarcomas: a randomized comparative trial. A phase III, Southwest Oncology Group Study (7613).. J Clin Oncol.

[OCR_00305] Benjamin R. S. (1987). Grade 3 nausea, vomiting, and myelosuppression or progressive, metastatic sarcoma?. J Clin Oncol.

[OCR_00319] Bodey G. P., Rodriguez V., Murphy W. K., Burgess A., Benjamin R. S. (1981). Protected environment - prophylactic antibiotic program for malignant sarcomas: randomized trial during remission induction chemotherapy.. Cancer.

[OCR_00323] Borden E. C., Amato D. A., Rosenbaum C., Enterline H. T., Shiraki M. J., Creech R. H., Lerner H. J., Carbone P. P. (1987). Randomized comparison of three adriamycin regimens for metastatic soft tissue sarcomas.. J Clin Oncol.

[OCR_00328] Brade W. P., Herdrich K., Varini M. (1985). Ifosfamide--pharmacology, safety and therapeutic potential.. Cancer Treat Rev.

[OCR_00333] Bramwell V. H. (1988). Current perspectives in the management of soft-tissue sarcoma. The role of chemotherapy in multimodality therapy.. Can J Surg.

[OCR_00338] Bramwell V. H., Mouridsen H. T., Mulder J. H., Somers R., Van Oosterom A. T., Santoro A., Thomas D., Sylvester R., Markham D. (1983). Carminomycin vs adriamycin in advanced soft tissue sarcomas: an EORTC randomised phase II study.. Eur J Cancer Clin Oncol.

[OCR_00344] Bramwell V. H., Mouridsen H. T., Santoro A., Blackledge G., Somers R., Verwey J., Dombernowsky P., Onsrud M., Thomas D., Sylvester R. (1987). Cyclophosphamide versus ifosfamide: final report of a randomized phase II trial in adult soft tissue sarcomas.. Eur J Cancer Clin Oncol.

[OCR_00350] Bryant B. M., Wiltshaw E. (1980). Results of the Royal Marsden Hospital second soft tissue sarcoma schedule (STS II) chemotherapy regimen in the management of advanced sarcoma.. Cancer Treat Rep.

[OCR_00356] Bui N. B., Chauvergne J., Hocke C., Durand M., Brunet R., Coindre J. M. (1985). Analysis of a series of sixty soft tissue sarcomas in adults treated with a cyclophosphamide-vincristine-adriamycin-dacarbazine (CYVADIC) combination.. Cancer Chemother Pharmacol.

[OCR_00363] Cantwell B. M., Carmichael J., Ghani S., Harris A. L. (1988). A phase II study of ifosfamide/mesna with doxorubicin for adult soft tissue sarcoma.. Cancer Chemother Pharmacol.

[OCR_00369] Chan H. S., Thorner P. S., Haddad G., Ling V. (1990). Immunohistochemical detection of P-glycoprotein: prognostic correlation in soft tissue sarcoma of childhood.. J Clin Oncol.

[OCR_00375] Cormier W. J., Hahn R. G., Edmonson J. H., Eagan R. T. (1980). Phase II study in advanced sarcoma: randomized trial of pyrazofurin versus combination cyclophosphamide, doxorubicin, and cis-dichlorodiammineplatinum(II) (CAP).. Cancer Treat Rep.

[OCR_00382] Edmonson J. H., Hahn R. G., Schutt A. J., Bisel H. F., Ingle J. N. (1983). Cyclophosphamide, doxorubicin, and cisplatin combined in the treatment of advanced sarcomas.. Med Pediatr Oncol.

[OCR_00388] Edmonson J. H., Long H. J., Richardson R. L., Creagan E. T., Green S. J. (1985). Phase II study of a combination of mitomycin, doxorubicin and cisplatin in advanced sarcomas.. Cancer Chemother Pharmacol.

[OCR_00396] Elias A., Ryan L., Sulkes A., Collins J., Aisner J., Antman K. H. (1989). Response to mesna, doxorubicin, ifosfamide, and dacarbazine in 108 patients with metastatic or unresectable sarcoma and no prior chemotherapy.. J Clin Oncol.

[OCR_00400] Fahrer C., Brachmann R., von der Helm K. (1989). Expression of c-sis and other cellular proto-oncogenes in human sarcoma cell lines and biopsies.. Int J Cancer.

[OCR_00405] Gerlach J. H., Bell D. R., Karakousis C., Slocum H. K., Kartner N., Rustum Y. M., Ling V., Baker R. M. (1987). P-glycoprotein in human sarcoma: evidence for multidrug resistance.. J Clin Oncol.

[OCR_00421] Gottlieb J. A., Benjamin R. S., Baker L. H., O'Bryan R. M., Sinkovics J. G., Hoogstraten B., Quagliana J. M., Rivkin S. E., Bodey G. P., Rodriguez V. (1976). Role of DTIC (NSC-45388) in the chemotherapy of sarcomas.. Cancer Treat Rep.

[OCR_00426] Hilgard P., Herdrich K., Brade W. (1983). Ifosfamide--current aspects and perspectives.. Cancer Treat Rev.

[OCR_00433] Karakousis C. P., Dal Cin P., Turc-Carel C., Limon J., Sandberg A. A. (1987). Chromosomal changes in soft-tissue sarcomas. A new diagnostic parameter.. Arch Surg.

[OCR_00436] Karakousis C. P., Rao U., Park H. C. (1982). Combination chemotherapy (CYVADIC) in metastatic soft tissue sarcomas.. Eur J Cancer Clin Oncol.

[OCR_00446] Lynch G., Magill G. B., Sordillo P., Golbey R. B. (1982). Combination chemotherapy of advanced sarcomas in adults with "CYOMAD" (S7).. Cancer.

[OCR_00451] Mansi J. L., Fisher C., Wiltshaw E., MacMillan S., King M., Stuart-Harris R. (1988). A phase I-II study of ifosfamide in combination with adriamycin in the treatment of adult soft tissue sarcoma.. Eur J Cancer Clin Oncol.

[OCR_00457] Mouridsen H. T., Bastholt L., Somers R., Santoro A., Bramwell V., Mulder J. H., van Oosterom A. T., Buesa J., Pinedo H. M., Thomas D. (1987). Adriamycin versus epirubicin in advanced soft tissue sarcomas. A randomized phase II/phase III study of the EORTC Soft Tissue and Bone Sarcoma Group.. Eur J Cancer Clin Oncol.

[OCR_00464] O'Bryan R. M., Luce J. K., Talley R. W., Gottlieb J. A., Baker L. H., Bonadonna G. (1973). Phase II evaluation of adriamycin in human neoplasia.. Cancer.

[OCR_00468] Pfeffer M. R., Sulkes A., Biran S. (1988). Cyclophosphamide, adriamycin, DTIC and vincristine with methotrexate in the treatment of advanced soft tissue sarcomas.. Isr J Med Sci.

[OCR_00474] Pinedo H. M., Bramwell V. H., Mouridsen H. T., Somers R., Vendrik C. P., Santoro A., Buesa J., Wagener T., van Oosterom A. T., van Unnik J. A. (1984). Cyvadic in advanced soft tissue sarcoma: a randomized study comparing two schedules. A study of the EORTC Soft Tissue and Bone Sarcoma Group.. Cancer.

[OCR_00480] Presgrave P., Woods R. L., Tattersall M. H., Coates A. S., Levi J. A., Fox R. M., Hedley D. (1987). Chemotherapy of adult soft tissue sarcoma with combination of doxorubicin and methotrexate.. Cancer Treat Rep.

[OCR_00496] Schoenfeld D. A., Rosenbaum C., Horton J., Wolter J. M., Falkson G., DeConti R. C. (1982). A comparison of adriamycin versus vincristine and adriamycin, and cyclophosphamide versus vincristine, actinomycin-D, and cyclophosphamide for advanced sarcoma.. Cancer.

[OCR_00503] Schütte J., Mouridsen H. T., Stewart W., Santoro A., van Oosterom A. T., Somers R., Blackledge G., Verweij J., Dombernowsky P., Thomas D. (1990). Ifosfamide plus doxorubicin in previously untreated patients with advanced soft tissue sarcoma. The EORTC Soft Tissue and Bone Sarcoma Group.. Eur J Cancer.

[OCR_00514] Subramanian S., Wiltshaw E. (1978). Chemotherapy of sarcoma. A comparison of three regimens.. Lancet.

